# Preclinical evaluation of avutometinib and defactinib in high‐grade endometrioid endometrial cancer

**DOI:** 10.1002/cam4.70210

**Published:** 2024-09-06

**Authors:** Tobias Max Philipp Hartwich, Miranda Mansolf, Cem Demirkiran, Michelle Greenman, Stefania Bellone, Blair McNamara, Shuvro P. Nandi, Ludmil B. Alexandrov, Yang Yang‐Hartwich, Silvia Coma, Jonathan Pachter, Alessandro D. Santin

**Affiliations:** ^1^ Department of Obstetrics, Gynecology, and Reproductive Sciences Yale University New Haven Connecticut USA; ^2^ Department of Cellular and Molecular Medicine University of California San Diego La Jolla California USA; ^3^ Verastem Oncology Needham Massachusetts USA

**Keywords:** avutometinib, defactinib, endometrial cancer, FAK inhibitor, MEK inhibitor

## Abstract

**Background:**

High‐grade endometrial cancers (EAC) are aggressive tumors with a high risk of progression after treatment. As EAC may harbor mutations in the RAS/MAPK pathways, we evaluated the preclinical in vitro and in vivo efficacy of avutometinib, a RAF/MEK clamp, in combination with the focal adhesion kinase (FAK) inhibitors defactinib or VS‐4718, against multiple primary EAC cell lines and xenografts.

**Methods:**

Whole‐exome sequencing (WES) was used to evaluate the genetic landscape of five primary EAC cell lines. The in vitro activity of avutometinib and defactinib as single agents and in combination was evaluated using cell viability, cell cycle, and cytotoxicity assays. Mechanistic studies were performed using Western blot assays while in vivo experiments were completed in UTE10 engrafted mice treated with either vehicle, avutometinib, VS‐4718, or their combination through oral gavage.

**Results:**

WES results demonstrated multiple EAC cell lines to harbor genetic derangements in the RAS/MAPK pathway including KRAS/PTEN/PIK3CA/BRAF/ARID1A, potentially sensitizing to FAK and RAF/MEK inhibition. Five out of five of the EAC cell lines demonstrated in vitro sensitivity to FAK and/or RAF/MEK inhibition. By Western blot assays, exposure of EAC cell lines to defactinib, avutometinib, and their combination demonstrated decreased phosphorylated FAK (p‐FAK) as well as decreased p‐MEK and p‐ERK. In vivo the combination of avutometinib/VS‐4718 demonstrated superior tumor growth inhibition compared to single‐agent treatment and controls starting at Day 9 (*p* < 0.02 and *p* < 0.04) in UTE10 xenografts.

**Conclusions:**

Avutometinib, defactinib, and to a larger extent their combinations, demonstrated promising in vitro and in vivo activity against EAC cell lines and xenografts. These preclinical data support the potential clinical evaluation of this combination in high‐grade EAC patients.

## 1 INTRODUCTION

Endometrial cancer [EAC] is one of the most common cancers afflicting women, ranking at sixth place in global incidence.^1^ In the United States alone, an estimated ~66,000 cases were diagnosed and about ~13,000 women died from this disease in 2023.^2^ Both the incidence rate as well as the mortality are increasing yearly, with the incidence showing a growth of 2% per year for women under the age of 50 and 1% for women older than 50, and an average increase in mortality of 0.7% each year from 2016 to 2020.^2^ While the majority of cases are caught at an early stage, about 31% of cases are diagnosed at more advanced stages.^2^ Poorly differentiated, high‐grade endometrioid endometrial tumors (G3‐EEC), similar to other biologic aggressive histologic types such as serous papillary and clear cell EACs are usually not associated with hyperestrogenic factors,^3^ are often deeply invasive in the myometrium and/or metastatic at presentation and often recur despite aggressive clinical interventions. Immunotherapy as monotherapy and/or in combination with chemotherapy showed improved outcomes in recent studies.^4^, ^5^ Unfortunately, no effective treatment options are currently available in the recurrent setting when poorly differentiated EAC acquire resistance to chemotherapy and/or immunotherapy.

The RAS/RAF/MEK/ERK (RAS/MAPK) pathway is involved in many vital cellular functions, including cell proliferation, gene expression, apoptosis, and cell survival. It is often activated or over‐expressed in many human solid tumors including endometrial cancer and thus represents a promising target for inhibitor treatments.^6^ Avutometinib is a novel RAF/MEK clamp that inhibits MEK kinase activity and also blocks the ability of RAF (ARAF, BRAF, and CRAF) to phosphorylate MEK.^7^ A known mechanism of adaptive resistance to RAF/MEK‐based treatment is activation of the cytoplasmic tyrosine kinase focal adhesion kinase (FAK).^8^, ^9^ Defactinib and VS‐4718^10^ (surrogate for defactinib in nonclinical mouse studies) are FAK inhibitors that have shown synergistic antitumor activity in combination with avutometinib in human cancer models by blocking this known mechanism of adaptive resistance to RAS/MAPK pathway inhibition via FAK activation.^11^, ^12^

As scant information is currently available on the potential activity of avutometinib and/or defactinib in EAC, in this study we analyzed the mutational signatures of five recently established and characterized primary EAC cell lines by whole‐exome sequencing (WES) and evaluated the preclinical activity of avutometinib and/or defactinib and VS‐4718 against EAC cell lines and xenografts. We provide the first experimental evidence to demonstrate that EAC harboring RAS/MAPK pathway alterations may be sensitive to RAF/MEK and FAK inhibitors both in vitro as well as in vivo.

## 2 MATERIALS AND METHODS

### 2.1 Establishment of primary uterine endometrioid cancer cell lines

Briefly, study approval was obtained from the Institutional Review Board (IRB). Prior to surgical staging and/or biopsy patients were consented for tumor banking in accordance with the Declaration of Helsinki. Five primary uterine endometrioid cell lines were established from patients at the time of primary surgical staging after sterile processing of fresh tumor biopsy samples, as previously described.^13^ Tissue source and cell line characteristics of the fully sequenced primary EAC cell lines used in our experiments are described in Table 1. Tumors were staged according to the 2009 International Federation of Gynecology and Obstetrics (FIGO) staging system.^14^ All primary EAC cells used in the experiments described below were performed with cell lines with limited passages (i.e., <50).

**TABLE 1:** Patient characteristics.

	Age	Ethnicity	Histology	Grade	FIGO stage
UTE1	80	White	Endometrioid endometrial	G3	IIIC1
UTE2	64	White	Endometrioid endometrial	G2	IB
UTE3	66	African–American	Endometrioid endometrial	G3	IIIA
UTE10	50	White	Mixed endometrioid and clear cell	G3	IIIA
UTE11	70	White	Endometrioid endometrial	G3	IIIC1

### 2.2 Whole‐exome sequencing

Briefly, DNA was extracted from tumor cell lines and when available, matching normal DNA was extracted from PBMC cells that were obtained from patients' blood collected at the same time as tumor samples. WES was performed as previously described.^15^ Briefly, DNA samples were processed at the Yale Center for Genome Analysis. Resulting unaligned fastq read files were analyzed according to GATK best practices (GATK version 4.4.0.0).^16^ Copy number variation was determined using FACETS.^17^, ^18^ Mutational signatures were extracted using SigProfilerExtractor^19^ as described before.^20^

### 2.3 Drugs

Both defactinib and VS‐4718 (FAKi) and avutometinib (RAF/MEKi) were obtained from Verastem Oncology through a material transfer agreement (MTA). Defactinib and avutometinib were dissolved in dimethyl sulfoxide (DMSO, Sigma‐Aldrich, St. Louis, MO) as a 10‐mM stock solution for the in vitro experiment. For in vivo experiments, avutometinib was prepared in 5% DMSO +10% hydroxypropyl‐3‐cyclodextrin (HPCD) in sterile water while VS‐4718 was prepared in 0.5% carboxymethyl cellulose (CMC) (C5678, Sigma‐Aldrich; St. Louis, MO) + 0.1% Tween 80 (P1754, Sigma‐Aldrich; St. Louis, MO) in sterile water (B. Braun Medical; Irvine, CA or equivalent).

### 2.4 Primary cell lines mutational signatures

WES data were analyzed for their mutational signatures as described by Alexandrov et al.^21^, ^22^ Briefly, mutational signatures were extracted using base substitutions and additionally included information on the sequence context of each mutation. Briefly, as there are six classes of base substitution C > A, C > G, C > T, T > A, T > C, T > G (all substitutions are referred to by the pyrimidine of the mutated Watson–Crick base pair) and as information on the bases immediately 5′ and 3′ to each mutated base is incorporated in this analysis, there are 96 possible mutations in this classification. In published studies, applying this approach to multiple human cancer types revealed over 30 distinct validated mutational signatures.^21^

### 2.5 Cell viability assay

Briefly, tumor cells were plated at a density of 60,000–150,000 cells/well in 6‐well tissue culture plates. After 24 h of incubation in RPMI 1640 media supplemented with 10% FBS, 1% penicillin/streptomycin, and 1% amphotericin, at 37°C, 5% CO_2_, cells were treated with defactinib or avutometinib at concentrations of 0, 0.001, 0.01, 0.1, 1, and 10 μM as single agents and their combinations and incubated for 72 h. After this treatment, cells were harvested in their entirety, stained with 10 μg/mL propidium iodide and the number of viable cells was then quantified using flow cytometry (BD FACSCalibur, BD Biosciences). Relative number of viable cells compared to untreated response as 100% were plotted as means ± standard deviations of at least three independent experiments, and half maximal inhibitory concentration (IC_50_) values were determined using GraphPad Prism 9. Synergy between avutometinib and defactinib during combination treatments was assessed by use of the software tool CompuSyn [ComboSyn Inc].

### 2.6 Western blot experiments

Cells were treated with control medium (RPMI 1640 with 0.1% DMSO), 1 μM avutometinib, 1 μM defactinib, or the combination of 1 μM avutometinib with 1 μM defactinib for 1 h. Cells were lysed using lysis buffer (1% Triton X, 0.05% SDS, 100 mM Na2HPO4, and 150 mM NaCl). Protein lysate was loaded onto 4%–20% pre‐cast SDS‐polyacrimide gels (Bio‐Rad) and transferred onto 0.45 PCDF Amersham Hybond membranes (GE Healthcare) after electrophoresis. Membranes were washed in PBS with 0.05% Tween 20 (PBST) and blocked with 5% milk in PBST. Primary antibody staining was done at 4°C overnight and secondary antibody staining after washing in PBST was done for 1 h. Chemiluminescence images were obtained using Clarity and Clarity Max ECL Western Blotting Substrates (Bio Rad) imaged on a digital Amersham Imager 680 (GE Healthcare Life Sciences).

### 2.7 Cell cycle

Cells were treated with avutometinib at concentrations of 0, 0.001, 0.01, 0.1, 1, and 10 μM for 24 h. Cells were collected in their entirety and fixed in 70% ethanol for 30 min. Cells were stained using 400 μL of propidium iodide (50 μg/mL) and imaged using flow cytometry. Cell cycle analysis was performed using software FlowJo version 9 (FlowJo, LLC).

### 2.8 Establishment of EAC xenografts

UTE10 primary cell line was used to establish tumor xenografts as previously described.^23^ In short, 200 μL of a 1:1 solution of sterile PBS and Matrigel (Corning Life Sciences) containing 10 million UTE10 cells were injected subcutaneously into the lower abdomen of female CB17/lcrHsd‐Prkdc/SCID mice. The developing tumor's size was measured three times per week using Vernier calipers and upon the tumor reaching about 0.25 cm^3^ in size, mice were randomized into the different treatment groups as described below.

### 2.9 In vivo experiments

Animals were randomized into treatment groups and five animals per cohort were then treated with either the vehicle (labeled “control”), 0.3 mg/kg avutometinib, 50 mg/kg VS‐4718, or combination (0.3 mg/kg avutometinib and 50 mg/kg VS‐4718). Treatments were administered for five consecutive days with 2 days of rest via oral gavage. Drugs were administered using 100 μL of vehicle as a single dose per treatment, except for the drug combination where animals were first treated with avutometinib and then 30 min later with VS‐4718. Mice were sacrificed upon reaching a tumor volume of 1 cm^3^ according to protocol approved by Yale's Institutional Animal Care and Use Committee.

### 2.10 Statistical analysis

GraphPad Prism 7 was used in statistical analysis (GraphPad Software, Inc. San Diego, CA). Ordinary two‐way ANOVA with uncorrected Fisher's LSD was used to determine the statistical significance of the effects of combination treatment on the different cell lines in vitro when compared to control and single‐agent treatments. Kaplan–Meier method was used to analyze overall survival data. Survival curves were compared using the log‐rank test. A two‐sided *p* < 0.05 is considered significant.

## 3 RESULTS

### 3.1 Genetic landscape of EAC

**FIGURE 1:**
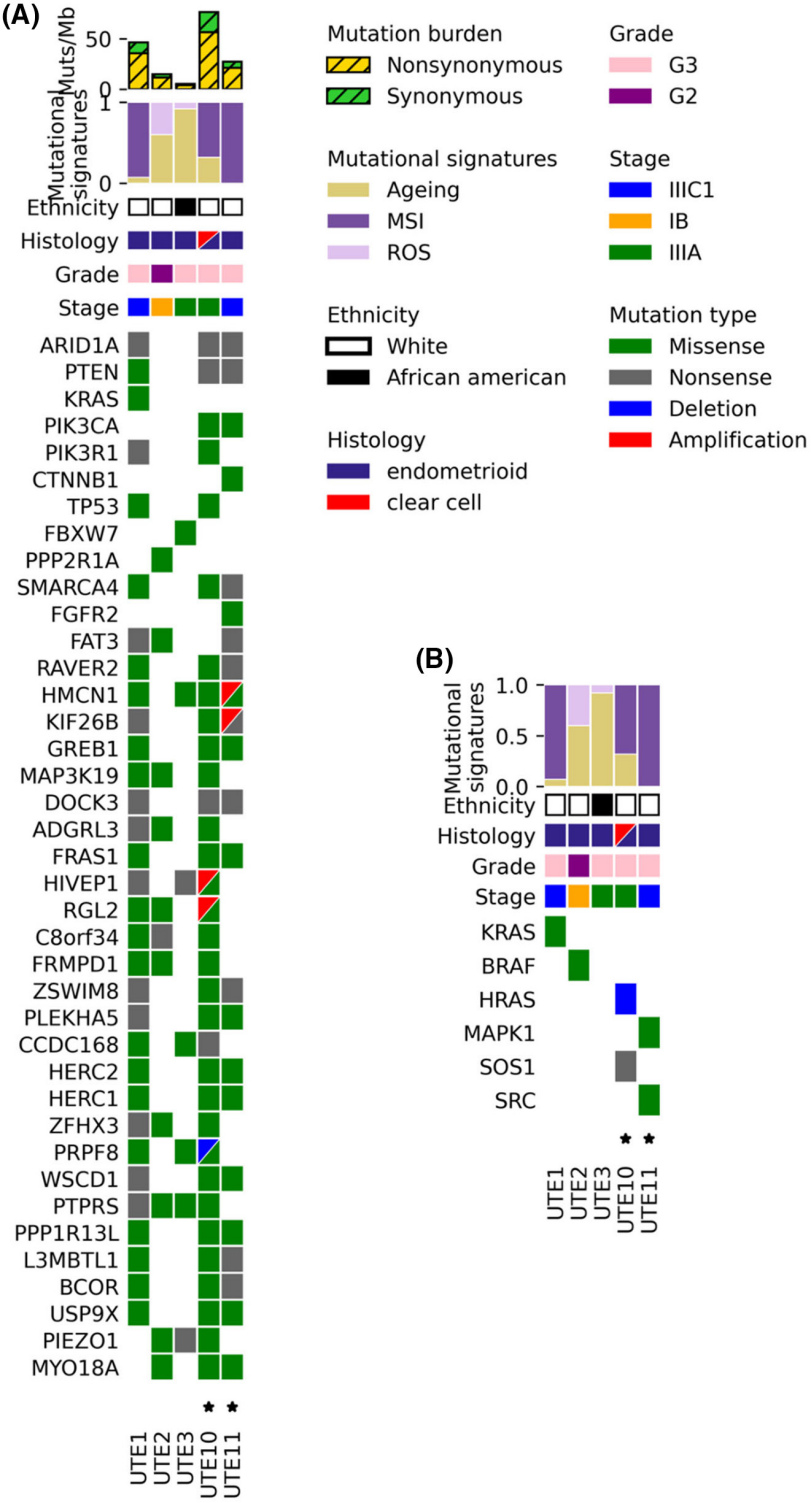
(A) Genetic landscape of five endometrioid endometrial cancer (EEC) cell lines showing tumor mutational burden, mutational signatures, clinical information, and alterations in genes associated with EC or present in at least three of the cell lines. (B) Genetic alterations in genes related to the RAS/MAPK pathway.

We sequenced five primary endometrial endometrioid cell lines using WES. Somatic gene alterations altered in at least three cell lines including mutated genes that have previously been associated as potential drivers in endometrioid endometrial cancers^24^ are shown in Figure 1. Tumor mutational burden (TMB) in the five cell lines for nonsilent mutations ranged from 3.9/Mb in UTE3 to 56.5/Mb in UTE10. Three out of five samples, UTE1, UTE10, and UTE11, showed a predominant mutational signature consistent with microsatellite instability (MSI). The remaining two samples, UTE2 and UTE3, had aging and ROS signatures (Figure 1). All five primary cell line samples showed multiple gene alterations previously associated with endometrioid tumors including ARID1A, PTEN, and SMARCA4 (Figure 1A). Except for UTE3, a primary tumor cell line characterized by a low TMB, the other four cell lines all harbored one or two alterations each in genes related to the RAS/MAPK pathway (i.e., the target of avutometinib) (Figure 1B) including a KRAS G13C alteration in UTE1 and a BRAF D594N alteration in UTE2.

### 3.2 In vitro antiproliferative activity of avutometinib ± defactinib

**FIGURE 2:**
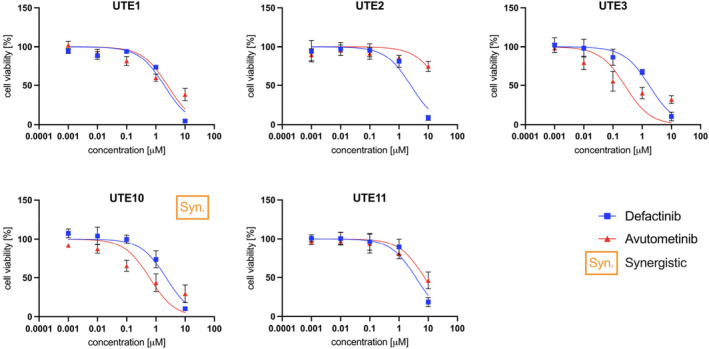
Effect of avutometinib and/or defactinib on cell viability. Cell lines were treated with varying doses of avutometinib, defactinib or their combination. UTE1, UTE3, UTE10, and UTE11 responded well to avutometinib treatment with IC_50_ values between 0.3 and 7.5 μM. UTE2 was resistant to avutometinib treatment and did not reach IC_50_. All five cell lines responded to defactinib with IC_50_ values between 1.7 and 3.8 μM. Only UTE10 showed synergistic effect of combination treatment.

We exposed the five primary EAC cell lines to avutometinib and defactinib as single agents and their combinations in vitro as described in the Materials and Methods section. All EAC cell lines responded to single‐agent treatment with defactinib showing decreasing viability with increasing dose of treatment. IC_50_ values for defactinib ranged from 1.7–3.8 ± 0.7 μM as shown in Table 2. Representative results are depicted in Figure 2. Four of the five cell lines responded to avutometinib with UTE3 and UTE10 demonstrating the higher sensitivity with IC_50_ values of 0.3 ± 0.1 μM and 0.6 ± 0.2 μM, respectively. UTE2 was the only cell line that did not reach an IC_50_ value after avutometinib exposure. Combination treatment with avutometinib plus defactinib demonstrated synergy at all tested concentrations only in the UTE10 cell line (Figure 2).

**TABLE 2:** Drug response (half maximal inhibitory concentrations, IC_50_).

	IC_50_ avutometinib	IC_50_ defactinib	Synergism
UTE1	2.5 ± 0.7 μM	1.9 ± 0.4 μM	–
UTE2	Not reached	2.5 ± 0.5 μM	–
UTE3	0.3 ± 0.1 μM	1.7 ± 0.3 μM	–
UTE10	0.6 ± 0.2 μM	2.2 ± 0.4 μM	Synergistic
UTE11	7.5 ± 1.2 μM	3.8 ± 0.7 μM	–

### 3.3 Avutometinib treatment induces cell cycle arrest

**FIGURE 3:**
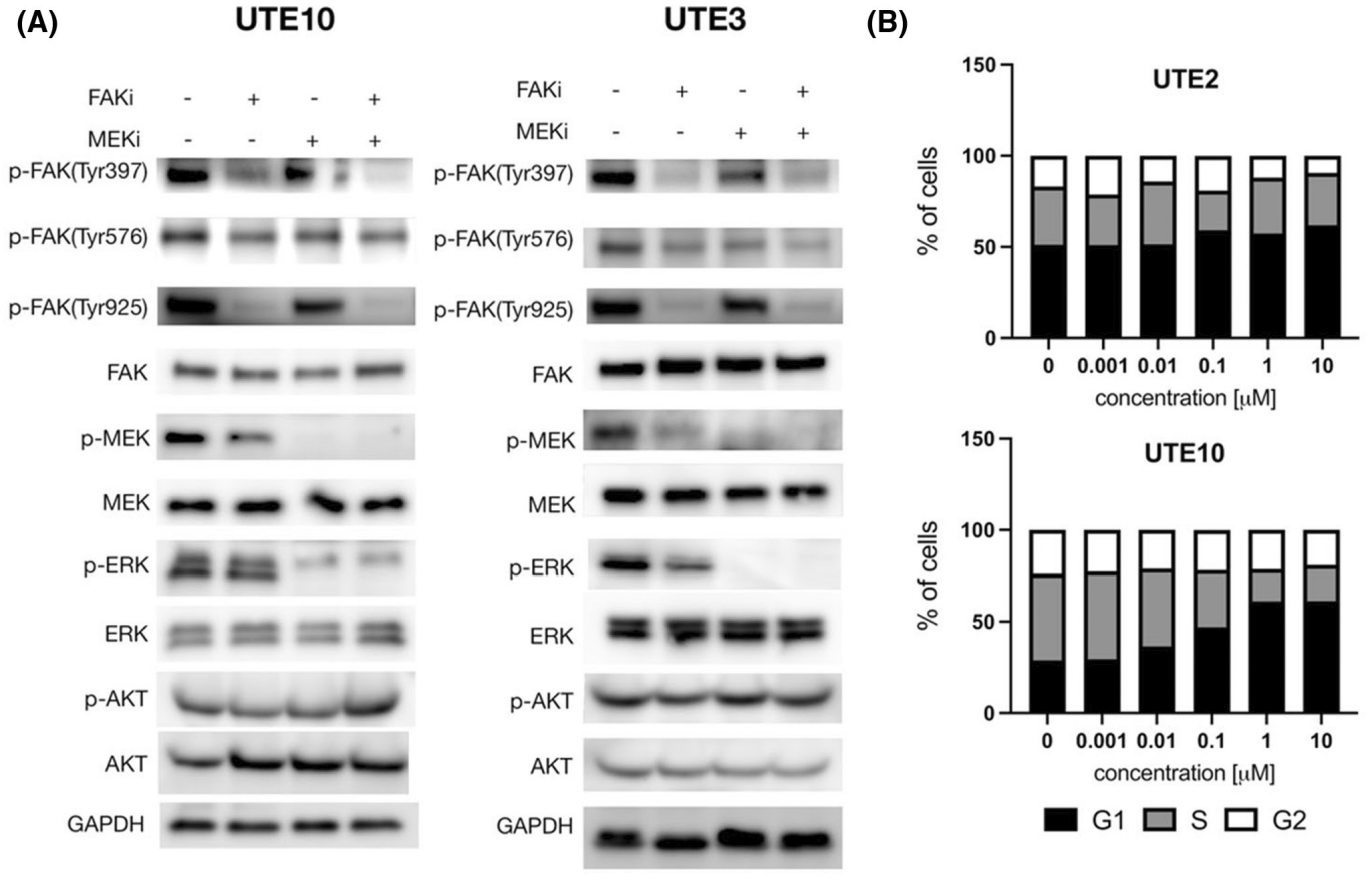
Western blot and cell cycle in EEC cell lines. (A) Representative images of western blot results for UTE10 (left) and UTE3 (right) treated with control, defactinib, avutometinib, and their combination. (B) Representative plots of cell cycle phase populations upon treatment with varying doses of avutometinib. FAKi: defactinib, MEKi: avutometinib.

We next investigated the effect of avutometinib treatment on the distribution of cell populations in the different cell cycle stages for both the resistant EAC cell line UTE2 as well as the sensitive cell line UTE10. UTE2 started with a relatively large fraction of cells in G1 stage and showed no significant changes in cell cycle stage populations upon treatment with avutometinib ranging from 0.001 to 10 μM. In contrast, UTE10 started with a lower percentage of cells in G1 stage and showed clear signs of cell cycle arrest with the population of cells in G1 phase increasing from about 30% in untreated control to about 60% in cells treated with 10 μM avutometinib (Figure 3B).

### 3.4 Avutometinib and defactinib treatment inhibits ERK and FAK activation

Western blotting was performed on tumor cells after 24 h of treatment with avutometinib, defactinib, and their combination at the selected concentrations as described in the Material and Methods section. Specifically, we evaluated protein expression levels of FAK, p‐FAK, MEK, p‐MEK, ERK, p‐ERK, AKT, and p‐AKT in UTE3 and UTE10, two of the EAC cell lines highly responsive to avutometinib (Figure 3A). When treated with single‐agent defactinib at 1 μM, both cell lines show clear inhibition of p‐FAK (Tyr397 and Tyr925), the intended target of defactinib. They also show a slight reduction of protein levels in p‐MEK. Single‐agent avutometinib treatment at 1 μM significantly reduced the protein levels of the intended target p‐MEK and p‐ERK while combination treatment with both avutometinib and defactinib at 1 and 1 μM respectively showed significant reduction in p‐FAK, p‐MEK, and p‐ERK (Figure 3A).

### 3.5 In vivo antitumor activity of avutometinib ± VS‐4718

**FIGURE 4:**
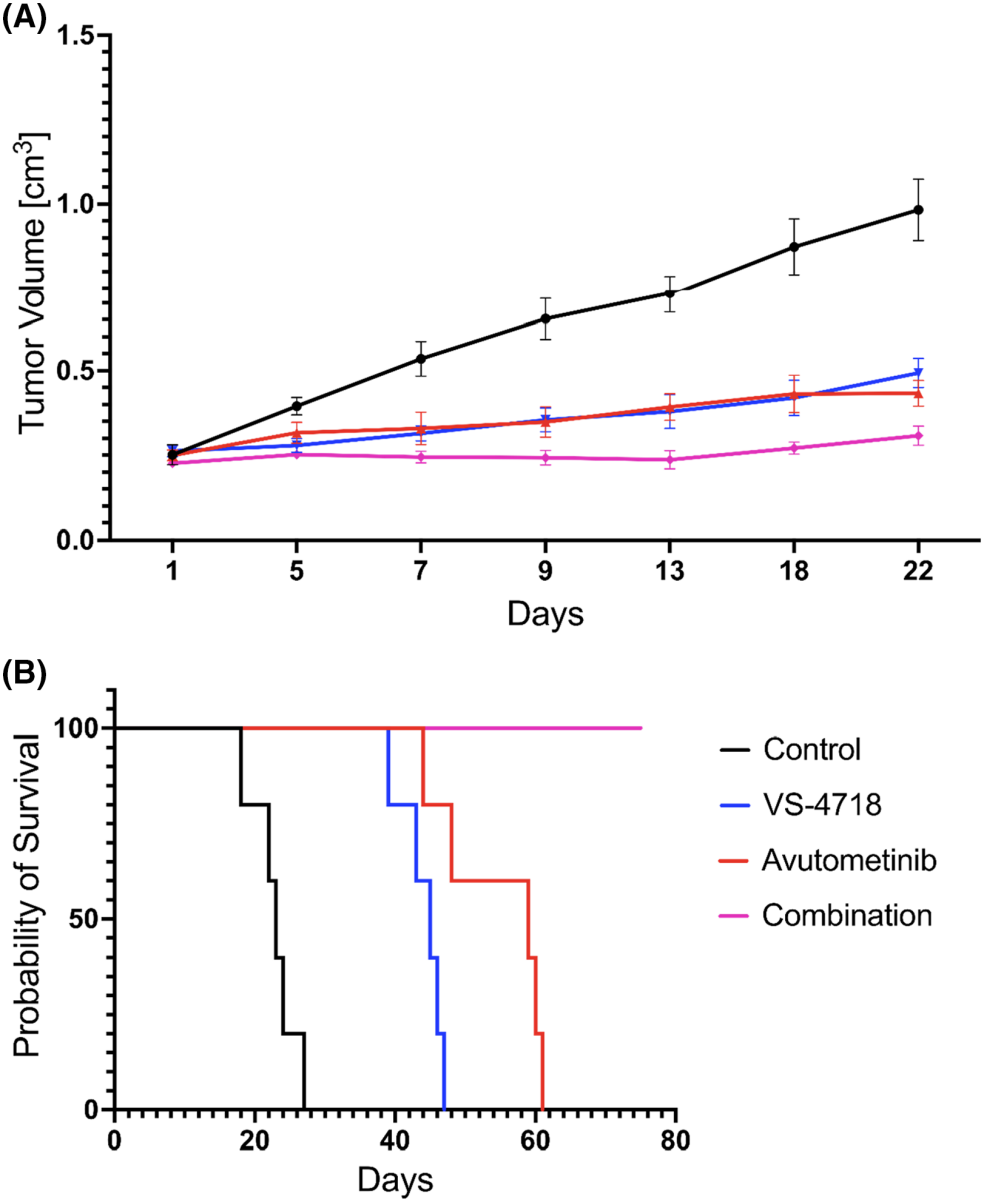
In vivo results in the UTE10 xenograft model. (A) Administration of avutometinib, defactinib, or combination treatment in daily oral doses, 5 days a week, all demonstrate growth inhibition compared to control. Tumor volume was significantly lower in mice treated with single avutometinib or defactinib versus control (*p* < 0.001, *p* < 0.001) and in combination versus control (*p* < 0.001) and versus either defactinib (*p* < 0.001) or avutometinib (*p* < 0.001). (B) Overall survival curves demonstrate the clear survival advantage of single‐agent avutometinib or defactinib treatments but especially of combination treatment. Median OS was 23 days for control, 55 days for defactinib treatment, 79 days for avutometinib treatment, and not reached after 75 days for combination treatment.

We evaluated the effects of avutometinib, VS‐4718 and their combination in vivo on a human endometrial cancer xenograft model generated using the UTE10 cell line. As shown in Figure 4, mice responded well to both single‐agent treatments and the drug combination. UTE10 tumor volume growth was significantly inhibited by single‐agent treatments versus control (*p* < 0.001) and to an even higher degree with the combination treatment (*p* < 0.001, Figure 4A). Combination treatment was also significantly more effective compared to either single‐agent avutometinib or defactinib treatments (*p* < 0.001 and *p* < 0.001, respectively). All animals in the treatment groups were still alive when the Control group reached the cutoff value of 1 cm^3^. The overall survival was 23 days for the control group, 45 days for the VS‐4718 group, and 59 days for the avutometinib group. None of the animals in the combination treatment groups died before the end of the study at Day 75 (Figure 4B).

## 4 DISCUSSION

Treatment with avutometinib, defactinib, and their combination demonstrated both in vitro and in vivo activity against primary endometrioid endometrial cancer cell lines and xenografts harboring alterations in the RAS/MAPK pathway. Four out of five EEC cell lines were sensitive to single‐agent avutometinib treatment, with IC_50_ values ranging between 0.3 ± 0.1 μM and 7.5 ± 1.2 μM while five out of five cell lines responded well to single‐agent treatment with defactinib with IC_50_ values ranging between 1.7 ± 0.3 μM and 3.8 ± 0.7 μM. The combination of avutometinib and defactinib was synergistic both in vitro and in vivo against UTE‐10, with treatment with avutometinib, VS‐4718 or their combination able to extend the median survival of the UTE‐10 engrafted animals from 23 days in the control group by a factor of 2.6‐fold, 2‐fold, and >3‐fold respectively.

While endometrial cancer (EC) is generally considered a gynecologic tumor with good prognosis and is diagnosed at early stages in about 69% of cases, 5‐year survival rates drop for later stage disease.^2^ Patients diagnosed with SEER stage “distant” disease have 5‐year OS of <20%.^25^ Unfortunately, for many of these patients, the prognosis remains poor, regardless of their treatment after surgery with gold standard adjuvant therapies including radiation, chemotherapy, and/or immunotherapy. The development of novel, effective treatment modalities against recurrent EAC resistant to standard treatments remains an unmet medical need.

Comprehensive next‐generation sequencing data from the tumor cancer genome atlas network (TCGA) and other research groups have recently demonstrated common mutations across EAC in multiple genes including KRAS, NRAS, and BRAF suggesting that the RAS/MAPK pathway may be critical to the pathogenesis of many EAC.^24^, ^26^, ^27^ Importantly, these findings have provided support to the notion that targeting tumors with a dysregulated RAS/MAPK pathway using specific small molecule inhibitors (i.e., MEKi) may represent a novel, potentially effective treatment against recurrent poorly differentiated EAC. Accordingly, in this study we took advantage of five whole‐exome‐sequenced primary uterine EAC cell lines to preclinically evaluate the in vitro and in vivo activity of avutometinib, a novel RAF/MEK clamp, in combination with the focal‐adhesion‐kinase (FAK) inhibitors defactinib or VS‐4718. We provide the first evidence demonstrating the preclinical efficacy of RAF/MEK and FAK inhibitors and their combination against multiple poorly differentiated (G3) EAC using both in vitro and in vivo models.

Out of the five primary EAC cell lines available to our study, the UTE2 cell line was the only cell line found resistant to avutometinib. While the reasons for the lack of in vitro response to the RAF/MEKi is not completely understood, it is worth noting that UTE2 was the only EAC showing a moderate degree of differentiation (i.e., G2) and the only primary tumor cell line established from a patient harboring early stage (i.e., IB) disease. Indeed, all remaining primary cell lines were derived from poorly differentiated (grade G3) tumors collected from patients with advanced stages cancers (i.e., Stages IIIA‐IIIC1). Consistent with its moderate histologic differentiation, UTE2 also demonstrated a slower cell growth in vitro, as indicated by the cell cycle data showing a larger fraction of cells residing in G1 phase when compared to G3 EAC (i.e., UTE10). By WES analysis UTE2 was found to harbor an inactivating BRAF D594N mutation.^28^ Of interest, in previous studies, this BRAF mutation has been linked to increased levels of phosphorylated MEK and ERK when additional activating RAS mutations are present, which we did not identify in UTE2, as this signaling is limited by the ERK‐dependent feedback inhibition of RAS.^29^ Accordingly, we speculated that secondary to the absence of RAS mutations, the lack of effectiveness of avutometinib in UTE2 may be due to an adaptation of this tumor to reduced RAS/MAPK pathway signaling (i.e., limited addiction/dependency to the pathway). In support of this view, in Phase II studies the treatment with MEK inhibitor trametinib did not show any clinical effect on gynecologic cancer patients harboring a BRAF D594N alteration.^30^

Western blot results for UTE10 and UTE3, two of the representative EAC cell lines found sensitive to avutometinib in vitro, demonstrated clear inhibition of p‐FAK, p‐MEK, and p‐ERK when treated with the combination of defactinib and avutometinib. While the single‐agent treatment showed the expected inhibition of their specific molecular targets, the combined treatment was able to simultaneously block both the FAK and RAS/MAPK pathways demonstrating a synergistic effect. Of interest UTE10, which responded well to avutometinib, showed accumulation of cell population in G1 phase with reduction in G2 and S phases with increasing doses of avutometinib. These results are similar to the ones reported for other MEK inhibitors including 9za, a dual MEK/PDK1 inhibitor,^31^ and TAK‐733^32^ against different human tumors. In contrast, as discussed above, UTE2, which was resistant in vitro to avutometinib exposure, demonstrated no change in the respective cell cycle phase populations regardless to the dose used in the experiments.

Finally, in vivo results against a poorly differentiated EAC xenograft (UTE10) demonstrated clear benefits of both single agent treatment with avutometinib or defactinib and an even better response to combination treatment, extending the overall survival 2‐fold in the case of defactinib, 2.6‐fold for avutometinib, and beyond 3‐fold for the combination treatment. The increased efficacy of the combination treatment in vivo is in line with the in vitro results for UTE10, showing clear activity of both drugs against the FAK and MEK pathways in the western blot data, demonstrating synergism when tumor cells were treated with the drug combinations, as well as with previous published results for mono and combination treatment with avutometinib and defactinib for xenografts of other cancer models, such as lung, uterine carcinosarcoma, and low‐grade ovarian cancer.^12^, ^33^, ^34^

While our study is the first to investigate the preclinical activity of the novel RAF/MEK clamp avutometinib, the focal adhesion kinase inhibitor defactinib or their combination against primary EEC cell lines and xenografts it has limitations. One of these is the relatively small sample size of primary EEC cell lines tested in vitro and in vivo. Furthermore, the ethnic diversity of the cell lines available to our study is low, as only one of the primary cell lines tested was derived from an African American patient with EEC and molecular classification^24^, ^35^ was not performed due to lack of necessary information. Among the strengths are the use of genetically fully sequenced primary tumor cell lines in both our in vitro and in vivo experiments. Another strength of our study is that four out of five cell lines demonstrated high‐grade (G3) EEC histology, which affects up to 35% of EC patients and is often associated with poor prognosis and outcome.

These data using biologically aggressive endometrial cancer models are consistent and extend the recent preclinically work from our group using these novel RAF/MEKi and FAKi against other difficult to treat gynecologic tumors (i.e., uterine carcinosarcomas) as well as the encouraging data recently presented in patient‐derived low‐grade serous ovarian cancer (LGSOC) using in vivo models treated with the combination avutometinib/defactinib.^12^, ^33^, ^34^ Consistent with this view, the combination of avutometinib with defactinib is currently being evaluated in a multicenter, randomized, open‐label, Phase 2 study of patients with molecularly profiled recurrent LGSOC in ENGOT‐ov60/GOG‐3052/RAMP 201 (NCT04625270). Interim analysis of part A demonstrated confirmed ORRs of 45% (13/29; 95% CI: 26%, 64%) and tumor shrinkage in the vast majority of LGSOC patients (86%; 25/29).^27^ A KRAS mutant responses of 60% (9/15) and a KRAS wild‐type responses of 29% (4/14) were observed. The majority of adverse events were Grades 1–2 and a limited number of patients experienced dose reductions or discontinuations. An international confirmatory Phase 3 study (GOG‐3097/ENGOT‐ov81/NCRI/RAMP 301; NCT06072781) comparing avutometinib + defactinib to investigator's choice of treatment in patients with recurrent LGSOC is now enrolling. Additionally, the combination of avutometinib with defactinib is now being evaluated in patients with other gynecological cancers (NCT05512208; NCT05787561). Taken together, these trials in LGSOC combined with our preclinical data in uterine cancer suggest that RAF/MEK/FAK inhibition may represent a novel strategy with potential clinical efficacy in other biologically aggressive tumors such as poorly differentiated EAC.

In conclusion, we report the significant preclinical activity of avutometinib in combination with FAK inhibition against primary EAC cell lines and xenografts. These data support that the combination of avutometinib with defactinib may represent a novel potentially effective combination for patients with EAC. The design and implementation of clinical trials with avutometinib plus defactinib in patients with gynecologic cancer harboring tumors addicted to alterations in the RAS/MAPK pathway such as recurrent chemotherapy‐resistant high‐grade endometrial cancers is warranted.

## AUTHOR CONTRIBUTIONS

**Tobias Max Philipp Hartwich:** Conceptualization (equal). **Miranda Mansolf:** Data curation (equal). **Cem Demirkiran:** Data curation (equal). **Michelle Greenman:** Conceptualization (equal); data curation (equal). **Stefania Bellone:** Conceptualization (equal); formal analysis (equal); methodology (equal). **Blair McNamara:** Conceptualization (equal); data curation (equal); writing – original draft (equal). **Shuvro P. Nandi:** Formal analysis (equal). **Ludmil B. Alexandrov:** Data curation (equal); software (equal); supervision (equal). **Yang Yang‐Hartwich:** Data curation (equal); supervision (equal); validation (equal). **Silvia Coma:** Conceptualization (equal); supervision (equal). **Jonathan Pachter:** Conceptualization (equal); supervision (equal). **Alessandro D. Santin:** Conceptualization (equal); data curation (equal); formal analysis (equal); funding acquisition (equal); investigation (equal); methodology (equal); project administration (equal); resources (equal); software (equal); supervision (equal); validation (equal); visualization (equal); writing – original draft (equal); writing – review and editing (equal).

## FUNDING INFORMATION

This work was supported in part by grants from NIH U01 CA176067‐01A1, the Deborah Bunn Alley Foundation, the Domenic Cicchetti Foundation, the Discovery to Cure Foundation and the Guido Berlucchi Foundation to A.D.S. This investigation was also supported by NIH Research Grant CA‐16359 from NCI and Verastem Oncology research grant to A.D.S.

## CONFLICT OF INTEREST STATEMENT

ADS reports grants from PUMA, GILEAD, SYNTHON, MERCK, BOEHINGER‐INGELHEIM, GENENTECH, VERASTEM, and personal fees for consulting services from TESARO, EISAI, GSK, MERCK, and GILEAD. SC and JP work for Verastem Oncology. LBA is a co‐founder, CSO, scientific advisory member, and consultant for io9, has equity, and receives income. The terms of this arrangement have been reviewed and approved by the University of California, San Diego, in accordance with its conflict of interest policies. LBA's spouse is an employee of Biotheranostics. LBA declares U.S. provisional applications with serial numbers: 63/289,601; 63/269,033; 63/483,237; 63/366,392; 63/412,835; and 63/492,348. The other authors declare no conflict of interest.

## ETHICS STATEMENT

This study was approved by Yale University HIC# 0804003759.

## Data Availability

Data are available within the article or its supplementary materials.
